# Coxsackievirus A16 in Southern Vietnam

**DOI:** 10.3389/fmicb.2021.689658

**Published:** 2021-06-24

**Authors:** Le Nguyen Truc Nhu, Le Nguyen Thanh Nhan, Nguyen To Anh, Nguyen Thi Thu Hong, Hoang Minh Tu Van, Tran Tan Thanh, Vu Thi Ty Hang, Do Duong Kim Han, Nguyen Thi Han Ny, Lam Anh Nguyet, Du Tuan Quy, Phan Tu Qui, Truong Huu Khanh, Nguyen Thanh Hung, Ha Manh Tuan, Nguyen Van Vinh Chau, Guy Thwaites, H. Rogier van Doorn, Le Van Tan

**Affiliations:** ^1^Oxford University Clinical Research Unit, Ho Chi Minh City, Vietnam; ^2^Children’s Hospital 1, Ho Chi Minh City, Vietnam; ^3^Children’s Hospital 2, Ho Chi Minh City, Vietnam; ^4^Hospital for Tropical Disease, Ho Chi Minh City, Vietnam; ^5^Centre for Tropical Medicine and Global Health, Nuffield Department of Medicine, University of Oxford, Oxford, United Kingdom

**Keywords:** coxsackievirus A16, hand foot mouth disease, picornavirus, evolution, Vietnam

## Abstract

**Background:** Hand, Foot and Mouth Disease (HFMD) is a major public health concern in the Asia-Pacific region. Most recent HFMD outbreaks have been caused by enterovirus A71 (EV-A71), coxsackievirus A16 (CVA16), CVA10, and CVA6. There has been no report regarding the epidemiology and genetic diversity of CVA16 in Vietnam. Such knowledge is critical to inform the development of intervention strategies.

**Materials and Methods:** From 2011 to 2017, clinical samples were collected from in- and outpatients enrolled in a HFMD research program conducted at three referral hospitals in Ho Chi Minh City (HCMC), Vietnam. Throat or rectal swabs positive for CVA16 with sufficient viral load were selected for whole genome sequencing and evolutionary analysis.

**Results:** Throughout the study period, 320 CVA16 positive samples were collected from 2808 HFMD patients (11.4%). 59.4% of patients were male. The median age was 20.8 months (IQR, 14.96–31.41). Patients resided in HCMC (55.3%), Mekong Delta (22.2%), and South East Vietnam (22.5%). 10% of CVA16 infected patients had moderately severe or severe HFMD. CVA16 positive samples from 153 patients were selected for whole genome sequencing, and 66 complete genomes were obtained. Phylogenetic analysis demonstrated that Vietnamese CVA16 strains belong to a single genogroup B1a that clusters together with isolates from China, Japan, Thailand, Malaysia, France and Australia. The CVA16 strains of the present study were circulating in Vietnam some 4 years prior to its detection in HFMD cases.

**Conclusion:** We report for the first time on the molecular epidemiology of CVA16 in Vietnam. Unlike EV-A71, which showed frequent replacement between subgenogroups B5 and C4 every 2–3 years in Vietnam, CVA16 displays a less pronounced genetic alternation with only subgenogroup B1a circulating in Vietnam since 2011. Our collective findings emphasize the importance of active surveillance for viral circulation in HFMD endemic countries, critical to informing outbreak response and vaccine development.

## Introduction

First described in 1957 (Esposito and Principi, 2018), Hand, Foot and Mouth Disease (HFMD) is a common childhood illness caused by a wide range of enteroviruses (EVs) of the genus *Enterovirus* of the family *Picornaviridae* (Cai et al., 2019). The disease is characterized with lesions on the skin and oral mucosa, and is generally self-limited within a few days. However, clinical complications may occur, and include encephalitis, myocarditis, aseptic meningitis, pulmonary edema, acute flaccid paralysis and even death (Chen Q. et al., 2019). HFMD is now endemic in the Asia-Pacific region. Currently, there is no effective antiviral to treat the severe patients, while only EV-A71vaccine has been successfully developed and used in Mainland China.

Historically, most of the HFMD outbreaks have been caused by EV-A71 and Coxsackievirus A16 (CVA16) (Cai et al., 2019). EV-A71 can be associated with severe clinical outcomes while CVA16 is responsible for milder illness. In recent years, CVA6 and CVA10 have also been recognized as emerging pathogens of HFMD. EV-A71 and CVA16 are phylogenetically closely related (Brown et al., 2020). Genetically, CVA16 is divided into 2 genogroups A and B, with genogroup B being further divided into B1a, B1b, B1c, and B2 (Noisumdaeng et al., 2019). Recently new genogroups (C and D) have been reported in Peru, France and China (Chen L. et al., 2019).

In Vietnam, the first HFMD outbreak was reported in 2005, predominantly caused by EV-A71 and CVA16 (Phan et al., 2007). In 2008, HFMD became a notifiable disease in Vietnam, and a major outbreak was recorded in 2011–2012 with over 200,000 children admitted to hospitals and more than 200 deaths (Van Hoang et al., 2019b). Although HFMD cases have been reported nationwide, the number of reported, severe and fatal cases is greater in southern regions of Vietnam.

There are limited data regarding the epidemiology and genetic diversity of CVA16 from Vietnam. Likewise, globally, only 200 complete genomes of CVA16 have been deposited in GenBank or Virus Pathogen resource database^1^. Hence, to inform the development and implementation of intervention strategies, especially HFMD vaccines, we studied the molecular epidemiology of CVA16 in Vietnam.

## Materials and Methods

### Study Program

Clinical samples used in this study were obtained from patients enrolled in a HFMD research program conducted at 3 referral hospitals in Ho Chi Minh City (HCMC), Viet Nam between 2011 and 2017 (Le et al., 2020). For the period between August 2011 and June 2013, the study was conducted in the pediatric intensive care unit (PICU) of the Hospital for Tropical Diseases (Geoghegan et al., 2015). Accordingly, only severe patients (i.e., those with grade 2b1 or above) were enrolled (grading system according to the guidelines issued by the Vietnamese Ministry of Health, Supplementary Table 1). In the second stage, July 2013–December 2017, the study sites were expanded to Children’s Hospital 1 and Children’s Hospital 2 and the recruitment covered patients seen in outpatient clinics, or admitted to the infectious disease wards or PICUs of these three study sites (Van Hoang et al., 2019a).

We collected information regarding demographic and clinical grades (Supplementary Table 1) on enrolment to the study and (if inpatients) daily until discharge or day 7 of hospitalization (whichever came first). Additionally, we sampled acute throat- and rectal swabs at enrolment from each participant. For the analysis of the present study, any throat or rectal swabs positive for CVA16 with a cycle threshold of ≤ 30 were selected for whole genome sequencing.

### Random PCR and Library Preparation

Whole genome sequencing of selected samples was performed using a previously described MiSeq-based approach (Nguyen et al., 2016). Briefly, 110μl of the selected swab in viral transport medium was centrifuged at 13,000 rpm for 10 min to remove host cells or large cell debris. The collected supernatants were treated with DNase at 37°C for 30 min. Viral nucleic acid was then extracted using QIAamp viral RNA kit (Qiagen, Hilden, Germany) and recovered in 50 μL elution buffer provided with the kit. cDNA was synthesized from 10 ul of viral nucleic acid using Super Script III enzyme (Invitrogen, Carlsbad, CA, United States) and FR26RV-Endoh primer. This was followed by the conversion of cDNA into double-stranded (ds) DNA using exo-Klenow (Invitrogen), and random amplification of the resulting dsDNA using Platinum PCR Supermix (Invitrogen) and FR20RV primer. PCR products were then purified using AMPure (Beckman Coulter, Indianapolis, IN, United States) and processed to the library preparation step using Nextera XT DNA kit (Illumina, San Diego, CA, United States), following the manufacturer’s instructions. Finally, the prepared library was sequenced using MiSeq reagent kit v3 (600 cycles, Illumina), which produced read lengths up to 2 × 300 bp, in a MiSeq platform (Illumina) (Nguyen et al., 2016).

### Sequence Assembly/Editing and Recombinant Detection

Genomic reads coming out of the MiSeq platform were trimmed to remove adaptors. Consensus sequences of CVA16 genomes were then generated using reference-mapping method. This step involved mapping of all reads from each sample to a reference genome (accession number: KX595295) and generating consensus sequences using default settings, and manual editing of the obtained consensuses using Geneious software package version 8.1.5 (Biomatters, Auckland, New Zealand). The obtained CVA16 sequences were submitted to the National Center for Biotechnology Information (GenBank accession numbers: MZ043537-MZ043565 and MW999251-MW999294).

For phylogenetic analysis, we retrieved all CVA16 genomes available from Virus pathogen database and analysis resource. This dataset also includes all CVA16 sequences available in GenBank, and 6 previously reported VP1 sequences from Vietnam (Phan et al., 2007) in 2005. Identical sequences were removed. Sequences alignment was carried out using MUSCLE method available in Geneious. We then applied RDP4 [Recombination Detection Program version 4 (Martin et al., 2015)] to detect any recombinants in the dataset. Potential recombinants were confirmed by at least 3 of 4 selected methods, including Chimera, GENECONV, Maxchi, Bootscan, and Siscan (using default settings) with *p*-value < 0.05. Recombinant strains were then removed from further analysis.

### Phylogenetic Analysis

The relatedness between the Vietnamese and global CVA16 strains was investigated by reconstructing maximum-likelihood phylogenetic trees for viral capsid protein 1 (VP1) and complete coding sequences (CDS) utilizing IQ-TREE software version 1.4.3 (L. T. Nguyen et al., 2015). For these analyses, we used general time reversible and Tamura-Nei 93 nucleotide substitution models, for CDS and VP1 dataset, respectively, with gamma distribution among sites (four rate categories), as suggested by the IQ-TREE software.

For phylogeographic and timescale phylogenetic investigations of the Vietnamese CVA16 strains, any sequences with insufficient temporal signal, indicated by TempEst v1.5 (Rambaut et al., 2016), were excluded from analysis. Time-scale phylogenetic analysis was performed for both the 68 CDS and 90 VP1 sequence datasets obtained from the present study using BEAST package v1.8.3 (Drummond and Rambaut, 2007). For phylogeographic analysis, since the VP1 dataset more widely represented for the geographic locations of the study participants, we first focused our analysis on the VP1 sequences. Accordingly, all Vietnamese sequences were divided into 4 regions, including HCMC, Mekong Delta (Long An, Ben Tre, Dong Thap, Kien Giang, An Giang, Soc Trang, and Ca Mau), South East area (Dong Nai, Tay Ninh, Binh Duong, Lam Dong, and Binh Thuan), and Central Vietnam (Binh Dinh). Running the analysis with individual provinces would lead to inconclusive results because of the small number of samples. Similar analysis was also carried out for the CDS dataset.

We used Tamura-Nei 93 (Tamura and Nei, 1993) nucleotide substitution model for VP1 dataset (general time reversible for CDS dataset (Lanave et al., 1984)), with gamma distribution and 4 rate categories, strict molecular clock model and Bayesian skyline plot (10 groups). We employed a 1,300 million step Bayesian Markov chain Monte Carlo implemented in BEAST package, sampling every 1,30,000 steps for CDS dataset (and 100 million sampling every 10,000 steps for VP1 dataset). Convergence was evaluated by Tracer v1.5.1 (Rambaut et al., 2018) with 10% burn-in and effective sample size above 150. Maximum-clade credibility (MCC) trees were summarized with TreeAnnotator (BEAST package) and visualized in Figtree version 1.4.2 (Drummond and Rambaut, 2007).

#### Standard Biosecurity and Institutional Safety Procedures

All laboratory procedures described in the present study were conducted in a laboratory compliant with Good Clinical Laboratory Practice and biosafety level-2 laboratory certified by Vietnamese Ministry of Health. Accordingly, clinical samples are stored in biosecurity complied BSL2 repository. And testing of clinical samples was conducted in ISO 15189 certified laboratory. A protocol biosafety risk assessment was conducted and a risk management strategy for each laboratory procedure was developed and implemented. Staff working in the laboratory attended annual refreshment training on institutional biosafety and biosecurity.

## Results

### Demographics

**FIGURE 1:**
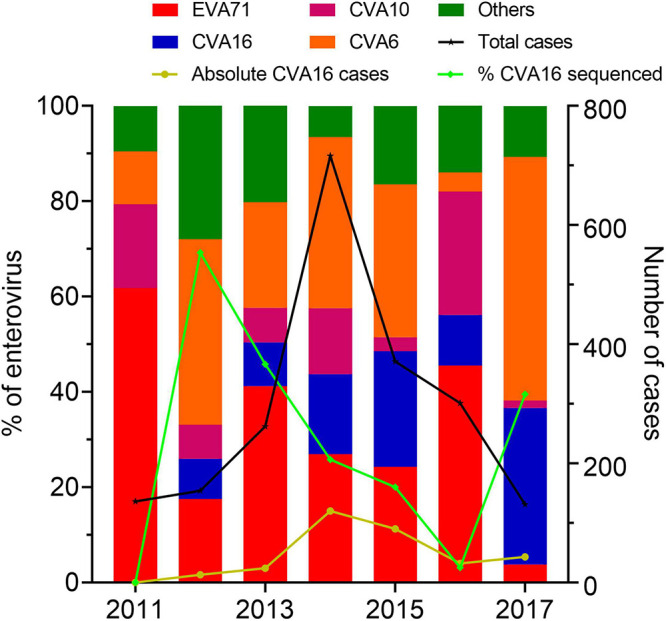
Enterovirus prevalence among HFMD cases in Vietnam during 2011–2017. The left *Y* axis shows the proportions of enterovirus serotypes detected among cases enrolled in the present study and the proportions of CVA16 cases included for whole genome sequencing. The right *Y* axis shows number of HFMD cases enrolled in the study between 2011 and 2017.

During 2011–2017, there were 2,813 HFMD patients enrolled in the study. Of these, 2,397 were positive for EV by real-time RT-PCR. CVA16 was detected in 322 patients, accounting for 13.43% of EV positive cases. Other common EVs detected included EV-A71, CVA6, and CVA10; accounting for 26.86, 24.53, and 10.18% of the PCR confirmed cases, respectively. During the study period, CVA16 was not detected in 2011, likely attributable the sampling bias of the study during this year. Then the proportion of CVA16 increased between 2014 and 2015 followed by a drop in 2016, and then increased again in 2017 (Figure 1).

320/322 CVA16 patients with available demographic and clinical information were included for analysis in the present study. Most (59.4%) of these patients were male, in agreement with previous reports (Le et al., 2020; Min et al., 2021). The median age was 20.82 months. 55.3% of the 320 patients resided in HCMC, the rest in Mekong Delta (22.2%) and South East Vietnam (22.5%). 32/320 (10%) of the CVA16 infected patients had moderately severe or severe (grade 2b1 and above) HFMD (Table 1).

**TABLE 1:** Demographic information and clinical grades of CVA16 cases in Vietnam 2011–2017.

		**Total (*n* = 320)**	**Sequenced group (*n* = 96)**	**Not sequenced group (*n* = 224)**	*p*-value*
Gender	Male	190 (59.4%)	54 (56.3%)	136 (60.7%)	0.46
	Female	130 (40.6%)	42 (43.8%)	88 (39.3%)	
Age (months)	Median	20.82	20.93	20.43	0.86
	IQR	14.96–31.41	14.23–33.71	15.23–29.4	
Provinces	HCMC	177 (55.3%)	47 (49.0%)	130 (58.0%)	0.42
	Mekong delta	71 (22.2%)	24 (25.0%)	47 (21.0%)	
	South East	71 (22.2%)	25 (26.0%)	46 (20.5%)	
	Central	1 (0.3%)	0 (0.0%)	1 (0.3%)	
Highest grade	1	152 (47.5%)	45 (46.9%)	107 (47.8%)	0.08
	2a	136 (42.5%)	36 (37.5%)	100 (44.6%)	
	2b1	20 (6.3%)	8 (8.3%)	12 (5.4%)	
	2b2	2 (0.6%)	2 (2.1%)	0 (0.0%)	
	3	10 (3.1%)	5 (5.2%)	5 (2.2%)	

Data on outcome could not be retrieved for severe patients of stage 1, 2011–2012 periods, so outcome comparison was not performed since it was likely to be biased. *Comparisons between sequenced and not sequenced groups. Fisher or Wilcoxon rank-sum test was used to compared between groups when appropriate.

### Phylogenetics

#### Results of Whole Genome Sequencing and Recombination Assessment

Of the 320 CVA16 patients included for analysis, 153 had a throat or rectal swab with sufficient viral load (Cp ≤ 30) for whole genome sequencing. Subsequently, 96 VP1 sequences were successfully recovered, including 81 from patients with mild (grade 1 or 2a) and 15 from those with severe diseases (grade 2b1, 2b2, or 3) (Table 1). Of these, 70 also had CDS successfully recovered. One CDS were indicated as recombinants (Supplementary Figure 1), and seven (6 VP1 and 1 CDS) sequences were identical sequences. We removed the identical sequences and recombinant sequences from subsequent analysis, and thus included 90 non-identical VP1 sequences and 68 CDS for detailed phylogenetic investigations.

#### Phylogenetic Analysis

**FIGURE 2:**
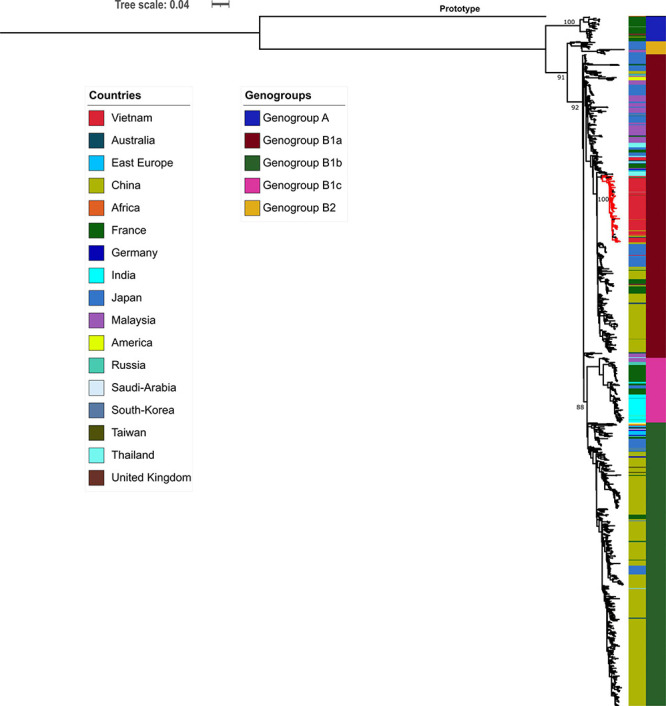
Maximum likelihood tree of CVA16 of Vietnam and global strains based on VP1 sequence. Vietnamese isolates (red-colored branch) belong to genogroup B1a containing strains from China, Thailand, Malaysia, Japan, France, and Germany.

Phylogenetic analyses of 1,066 global CVA16 VP1 sequences, including 90 Vietnamese sequences of the present study and 6 previously reported sequences from Vietnam in 2005, showed that CVA16 was divided into 2 genogroups, A and B, in accordance to previous studies (Perera et al., 2007; Noisumdaeng et al., 2018). Genogroup B subsequently split into B1 and B2 (subgroups a, b and c). All Vietnamese CVA16 sequences from this study belonged to group B1a, clustering together with strains collected in Vietnam in 2005, and from China, Thailand, Malaysia, Japan, France, and Germany (Figure 2).

#### Phylogeographics and Phylodynamics of CVA16 in Southern Vietnam

**FIGURE 3:**
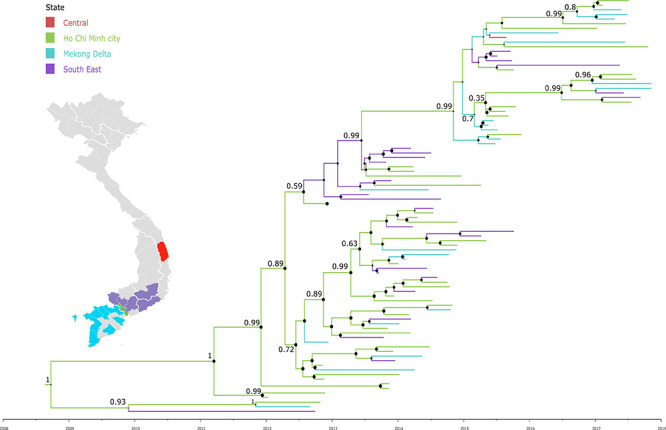
Maximum clade credibility trees demonstrating the phylogeography of CVA16 isolates in Vietnam. The tree was constructed using VP1 sequences of Vietnamese sequences and branches are colored by regions.

To investigate the dispersal of CVA16 in southern Vietnam, we performed detailed phylogeographic analysis for HCMC and other regions, including Mekong Delta, South East and Central Vietnam. The results revealed that CVA16 is dispersed widely across the southern regions of Vietnam during the study (Figure 3). Additionally, VP1-based time-scale phylogenetic analysis revealed that the time to most recent common ancestor of CVA16 sequences generated as part of the present study was estimated to be around the third quarter of 2008 (95% CI, April 2007–October 2009), 4 years before their first detection in HFMD cases enrolled in the clinical study from 2012 onward.

**FIGURE 4:**
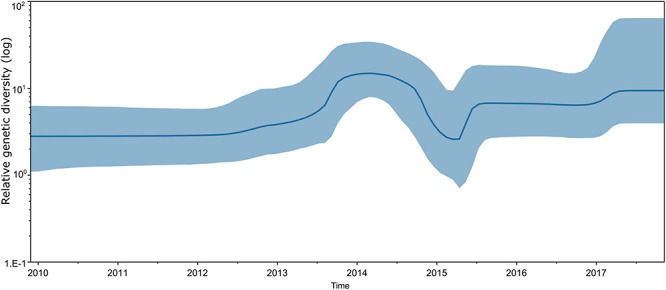
Estimated relative genetic diversity of CVA16 in Vietnam by Bayesian skyline plot using VP1 sequence dataset.

Bayesian skyline plot assessment revealed that the genetic diversity of CVA16 strains circulating in southern Vietnam remained stable during the study period (2011–2017), with some slight fluctuations corresponding to the increase in CVA16 cases reported in 2014–2015 and 2017 (Figure 4). The estimated evolution rate of CVA16 circulating in southern Vietnam was about 5.54 × 10^–3^ substitutions per site per year (95% CI, 4.57 × 10^–3^–6.59 × 10^–3^). Similar results of the time to most recent common ancestor, evolution rate and genetic diversity were obtained when the time-scale phylogenetic analysis was carried out for CDS dataset (Supplementary Figures 2, 3).

## Discussion

We report for the first time the detailed molecular epidemiology of CVA16 infection in Vietnam between 2011 and 2017. We showed that together with EV-A17, CVA10, and CVA6, CVA16 is one of the most predominant HFMD pathogens associated with severe clinical phenotypes in Vietnam during the study period (2011–2017). Of these, EV-A71 was the leading cause (Van Hoang et al., 2019a; Le et al., 2020). Additionally, despite its relatively stable genetic diversity, CVA16 dispersed widely between HCMC and other neighboring provinces. This reflected the endemicity of the pathogen and the burden posed by HFMD in southern Vietnam as a whole (Le et al., 2020, 2018).

A total of 32 CVA16 patients had severe HFMD (grade 2b1 or above), accounting for 10% of 320 CVA16 infected patients of the present study. While the data emphasize that CVA16 infection causes severe clinical consequences, this high proportion of clinical complications associated with CVA16 infection should be interpreted with caution. During the first stage of our study (2011–2012), we only focused our sampling on severe patients admitted to the PICU of the Hospital for Tropical Diseases. This group accounted for 12/32 (38%) of the severe cases of the present study. As such, our finding may have been biased by the over representation of the PICU admitted patients during the first phase of the study. Additionally, we were not able to include individuals with mild or asymptomatic infection who did not present to our study sites. As a consequence, our sampling biases hampered informative analysis aiming at identifying the association between viral strains and/or genetic variations and clinical severities. However, sub-analysis did not reveal such association in our dataset, supporting previous report (Singh et al., 2002; Shih et al., 2000).

Between 2011 and 2017, EV-A71 circulation was observed every 2 or 3 years (Van Hoang et al., 2019a; Le et al., 2020). Likewise, CVA6 and CVA16 replaced each other to become the dominant serotypes between the interval peaks of EV-A71. HFMD is a disease of young children, especially those without pre-existing immunity. Thus, the observed cyclic patterns of the common causes is likely attributable to the requirement of a sufficient number of susceptible newborn babies to sustain the ongoing transmission in the community.

EV-A71 subgenogroup replacements have frequently occurred in endemic countries (Le et al., 2018; Van Hoang et al., 2019a). In contrast, the CVA16 subgenogroup B1a was the only subgenogroup circulating in southern Vietnam during 2011–2017, and was genetically related to the B1a strains detected in HCMC in 2005, in Asia (China, Thailand, Malaysia and Japan) and in Europe (France and Germany). Together, the data pointed to the wide dispersal of B1a within Vietnam and globally. The genetic characteristics of CVA16 subgenogroup circulating in HCMC and Vietnam between 2006 and 2010 remain unknown.

Our estimated evolution rate for the VP1 dataset of the Vietnamese CVA16 sequences was 5.43 × 10^–3^ substitutions per site per year, supporting a recent report from China (Han et al., 2020), and our reported data (5.12 × 10^–3^ substitutions per site per year) for EV-A71 in Vietnam (Van Hoang et al., 2019a). In contrast, CVA6 showed slightly higher evolution rate (T. A. Nguyen et al., 2018), 7.42 × 10^–3^ substitutions per site per year. This might be partly explained by the more recent emergence of CVA6, compared to EV-A71 and CVA16, which warrants further investigations.

Our Bayesian-based analysis showed the most recent common ancestor (tMRCA) of CVA16 in Vietnam was dated to around August–September 2008. This suggested that CVA16 strains sequenced from patients enrolled in the present study had been circulating in Vietnam for 4 years before it was detected in HFMD cases enrolled in the present study from 2012 onward, supporting previous report regarding cryptic circulation of HFMD pathogens prior to causing community transmission (Tee et al., 2010). Recombination is a common phenomenon occurring as part of the evolutionary process of EVs (Simmonds and Welch, 2006). Likewise, we detected one recombinant CVA16 in our dataset. These collective findings in turn emphasize the importance of active surveillance for circulating HFMD strains in endemic countries, critical to informing public health authorities.

Our study had some limitations. As outlined above, from 2011 to 2012 our focus was patients admitted PICU of the Hospital for Tropical diseases. And we were not able to include individuals with mild or asymptomatic infection who did not present to our study sites. Therefore, we may have underestimated the diversity of CVA16 and EV serotypes detected in HFMD patients during this period, and have overestimated the frequency of severe manifestations from CVA16 infections. Additionally, the three hospitals in HCMC where our study was based are responsible for receiving HFMD from southern Vietnam. Therefore, while our findings may be generalized for southern Vietnam, the epidemiology of CVA16 in central and the north of Vietnam warrants further research.

In summary, our study demonstrates that CVA16 is an endemic HFMD pathogen with a single subgenogroup B1a circulating in southern Vietnam during 2011–2017. Despite its moderate evolution rate and stable genetic diversity, CVA16 was dispersed widely in southern Vietnam during the study period. Viral circulation in Vietnam happened some time in 2008, 4 years prior to its detection in HFMD cases enrolled in the present study from 2012 onward. These collective findings emphasize the importance of active surveillance for viral circulation in HFMD endemic countries, critical to informing outbreak response.

## Data Availability Statement

The data presented in the study are deposited in GenBank repository, accession numbers MZ043537 – MZ043565 and MW999227 – MW999294.

## Ethics Statement

The studies involving human participants were reviewed and approved by The Institutional Review Board of Children’s Hospital 1, Children’s Hospital 2 and Hospital for Tropical Diseases, and the Oxford Tropical Research Ethics Committee approved the study.

## Author Contributions

LNha, NH, HT, NC, GT, HD, and LT designed the study. HV, DH, DQ, PQ, TK, and LNha recruited the patients. LNhu, NA, NH, TT, VH, NN, and LN performed laboratory experiments. LNhu, NA, NH, and TT analyzed the data. LNhu drafted the manuscript. LT reviewed and edited the manuscript. All authors read the final manuscript and agreed with its contents.

## Conflict of Interest

The authors declare that the research was conducted in the absence of any commercial or financial relationships that could be construed as a potential conflict of interest.

## Footnotes

1
https://www.viprbrc.org

## References

[B1] Brown D. M. Zhang Y. Scheuermann R. H. (2020). Epidemiology and sequence-based evolutionary analysis of circulating non-polio enteroviruses. *Microorganisms* 8 1–23. 10.3390/microorganisms8121856 33255654 PMC7759938

[B2] Cai K. Wang Y. Guo Z. Yu H. Li H. Zhang L. (2019). Clinical characteristics and managements of severe hand, foot and mouth disease caused by enterovirus A71 and coxsackievirus A16 in Shanghai. *China. BMC Infect. Dis.* 19:285. 10.1186/s12879-019-3878-6 30917800 PMC6438032

[B3] Chen L. Yao X. J. Xu S. J. Yang H. Wu C. L. Lu J. (2019). Molecular surveillance of coxsackievirus A16 reveals the emergence of a new clade in mainland China. *Arch. Virol.* 164 867–874. 10.1007/s00705-018-4112-3 30498962

[B4] Chen Q. Zhang Q. Hu Z. (2019). Profiles of human enteroviruses associated with hand, foot, and mouth disease in nanjing, China. *Disaster Med. Public Health Prep.* 13 740–744. 10.1017/dmp.2018.155 30704549

[B5] Drummond A. J. Rambaut A. (2007). BEAST: bayesian evolutionary analysis by sampling trees. *BMC Evol. Biol.* 7:214. 10.1186/1471-2148-7-214 17996036 PMC2247476

[B6] Esposito S. Principi N. (2018). Hand, foot and mouth disease: Current knowledge on clinical manifestations, epidemiology, aetiology and prevention. *Eur. J. Clin. Microbiol. Infect. Dis.* 37 391–398. 10.1007/s10096-018-3206-x 29411190

[B7] Geoghegan J. L. Tan L. Van, Kühnert D. Halpin R. A. Lin X. Simenauer A. (2015). Phylodynamics of enterovirus A71-associated hand, foot, and mouth disease in viet nam. *J. Virol.* 89 8871–8879. 10.1128/jvi.00706-15 26085170 PMC4524079

[B8] Han Z. Song Y. Xiao J. Jiang L. Huang W. Wei H. (2020). Genomic epidemiology of coxsackievirus A16 in mainland of China, 2000–18. *Virus Evol.* 6 1–16. 10.1093/ve/veaa084 33343924 PMC7733612

[B9] Lanave C. Preparata G. Saccone C. Serio G. (1984). A new method for calculating evolutionary substitution rates. *J. Mol. Evol.* 20 86–93. 10.1007/bf02101990 6429346

[B10] Le N. T. N. Nguyen T. T. H. Le N. T. N. Lam A. N. Nguyen T. H. N. Tran T. T. (2018). Severe enterovirus A71 associated hand, foot and mouth disease. Vietnam, 2018: preliminary report of an impending outbreak. *Eurosurveillance* 23:1800590. 10.2807/1560-7917.ES.2018.23.46.1800590 30458911 PMC6247458

[B11] Le N. T. N. Truong H. K. Nguyen T. T. H. Van Hoang M. T. Le N. T. N. Nguyen T. H. N. (2020). Clinical, etiological and epidemiological investigations of hand, foot and mouth disease in Southern Vietnam during 2015 – 2018. *PLoS Negl. Trop. Dis.* 14:e0008544. 10.1371/journal.pntd.0008544 32804980 PMC7451980

[B12] Martin D. P. Murrell B. Golden M. Khoosal A. Muhire B. (2015). RDP4: detection and analysis of recombination patterns in virus genomes. *Virus Evol.* 1:vev003. 10.1093/ve/vev003 27774277 PMC5014473

[B13] Min N. Ong Y. H. B. Han A. X. Ho S. X. Yen E. W. P. Ban K. H. K. (2021). An epidemiological surveillance of hand foot and mouth disease in paediatric patients and in community: a singapore retrospective cohort study, 2013–2018. *PLoS Negl. Trop. Dis.* 15:e0008885. 10.1371/journal.pntd.0008885 33566802 PMC7901731

[B14] Nguyen L. T. Schmidt H. A. Von Haeseler A. Minh B. Q. (2015). IQ-TREE: a fast and effective stochastic algorithm for estimating maximum-likelihood phylogenies. *Mol. Biol. Evol.* 32 268–274. 10.1093/molbev/msu300 25371430 PMC4271533

[B15] Nguyen T. A. Le N. T. N. Hoang M. T. Van, Nguyen T. T. H. Tran T. T. (2018). Emerging coxsackievirus A6 causing hand. foot and mouth disease, vietnam. *Emerg. Infect. Dis.* 24 17–19. 10.3201/eid2404.171298 PMC5875260 29553326

[B16] Nguyen T. A. Tran T. T. Hoang M. T. Van, Nghiem M. N. Le N. T. N. (2016). Development and evaluation of a non-ribosomal random PCR and next-generation sequencing based assay for detection and sequencing of hand, foot and mouth disease pathogens. *Virol. J.* 13:125. 10.1186/s12985-016-0580-9 27388326 PMC4937578

[B17] Noisumdaeng P. Korkusol A. Prasertsopon J. Sangsiriwut K. Chokephaibulkit K. Mungaomklang A. (2019). Longitudinal study on enterovirus A71 and coxsackievirus A16 genotype/subgenotype replacements in hand, foot and mouth disease patients in Thailand, 2000–2017. *Int. J. Infect. Dis.* 80 84–91. 10.1016/j.ijid.2018.12.020 30639624

[B18] Noisumdaeng P. Sangsiriwut K. Prasertsopon J. Klinmalai C. Payungporn S. Mungaomklang A. (2018). Complete genome analysis demonstrates multiple introductions of enterovirus 71 and coxsackievirus A16 recombinant strains into Thailand during the past decade. *Emerg. Microbes Infect.* 7:1. 10.1038/s41426-018-0215-x 30552334 PMC6294798

[B19] Perera D. Yusof M. A. Podin Y. Ooi M. H. Thao N. T. T. Wong K. K. (2007). Molecular phylogeny of modern coxsackievirus A16. *Arch. Virol.* 152 1201–1208. 10.1007/s00705-006-0934-5 17308978

[B20] Phan V. T. Nguyen T. T. T. Perera D. Truong Th. K Nguyen T. K. T. Tang C. T. (2007). Epidemiologic and virologic investigation of hand, foot, and mouth disease, southern Vietnam, 2005. *Emerg. Infect. Dis.* 13 1733–1741. 18217559 10.3201/eid1311.070632 PMC3375788

[B21] Rambaut A. Drummond A. J. Xie D. Baele G. Suchard M. A. (2018). Posterior summarization in bayesian phylogenetics using tracer 1.7. *Syst. Biol.* 67 901–904. 10.1093/sysbio/syy032 29718447 PMC6101584

[B22] Rambaut A. Lam T. T. Max Carvalho L. Pybus O. G. (2016). Exploring the temporal structure of heterochronous sequences using tempEst (formerly Path-O-Gen). *Virus Evol.* 2:1. 10.1093/ve/vew007 27774300 PMC4989882

[B23] Shih S. R. Ho M. S. Lin K. H. Wu S. L. Chen Y. T. Wu C. N. (2000). Genetic analysis of enterovirus 71 isolated from fatal and non-fatal cases of hand, foot and mouth disease during an epidemic in Taiwan, 1998. *Virus Res.* 68 127–136. 10.1016/S0168-1702(00)00162-3 10958984

[B24] Simmonds P. Welch J. (2006). Frequency and dynamics of recombination within different species of human enteroviruses. *J. Virol.* 80 483–493. 10.1128/jvi.80.1.483-493.2006 16352572 PMC1317522

[B25] Singh S. Poh C. L. Chow V. T. K. (2002). Complete sequence analyses of enterovirus 71 strains from fatal and non-fatal cases of the hand, foot and mouth disease outbreak in Singapore (2000). *Microbiol. Immunol.* 46 801–808. 10.1111/j.1348-0421.2002.tb02767.x 12516778

[B26] Tamura K. Nei M. (1993). Estimation of the number of nucleotide substitutions in the control region of mitochondrial DNA in humans and chimpanzees. *Mol. Biol. Evol.* 10 512–526. 10.1093/oxfordjournals.molbev.a040023 8336541

[B27] Tee K. K. Lam T. T.-Y. Chan Y. F. Bible J. M. Kamarulzaman A. Tong C. Y. W. (2010). Evolutionary genetics of human enterovirus 71: origin, population dynamics, natural selection, and seasonal periodicity of the VP1 gene. *J. Virol.* 84 3339–3350. 10.1128/JVI.01019-09 20089660 PMC2838098

[B28] Van Hoang M. T. Nguyen T. A. Nguyen T. T. H. Le N. T. N. Lam A. N. Tran T. T. (2019a). Enterovirus A71 phenotypes causing hand, foot and mouth disease. Vietnam. *Emerg. Infect. Dis.* 25 788–791. 10.3201/eid2504.181367 30882309 PMC6433038

[B29] Van Hoang M. T. Nguyen T. A. Tran T. T. Vu T. T. H. Le N. T. N. Nguyen T. H. N. (2019b). Clinical and aetiological study of hand, foot and mouth disease in southern Vietnam, 2013–2015: inpatients and outpatients. *Int. J. Infect. Dis.* 80 1–9. 10.1016/j.ijid.2018.12.004 30550944 PMC6403263

