# Supplementary material for: Coxsackievirus A16 in Southern Vietnam
Source: Front Microbiol. 2021 Jun 24;12:689658. doi: 10.3389/fmicb.2021.689658 (PMC8265502; doi:10.3389/fmicb.2021.689658)
Supplement: Supplementary file 2 [file Data_Sheet_1.pdf]

Supplementary Materials

Supplementary Table 1: HFMD management guidelines according to Vietnamese Ministry of Health

	**Classification Signs/symptoms**	**Suggested Management**
Grade 1	Oral ulcers and/or vesicular rash on the hands, feet, and/or the buttocks	OPD care, with advice sheet for family<br>Careful observation for warning signs
Grade 2a	Grade 1 AND<br>Myoclonic jerks observed by the family (not witnessed by medical staff)<br>Legarthy, agitation/irritability<br>Fever ≥ 39°C for ≥ 48 hours. Vomiting	Hospitalization<br>Oral phenobarbitone<br>Vital Signs Monitoring: every 4-6 hours following standard protocols
Grade 2b1	Grade 1 AND<br>Myoclonic jerks witnessed by medical staff or by the family (2 jerks/30 minutes or 1 jerk and stupor)<br>Resting pulse rate > 130/min but <150/min (adjusted for fever")	Admit to HDU/PCIU<br>IV Phenobarbitone<br>Antipyretics<br>Vital Signs Monitoring: every 1-3 hours for minimum 6 hours until stable
Grade 2b2	Grade 1 AND witnessed myoclonic jerks accompanied by one of the following findings:<br>Continuous limb tremor, limb weakness<br>Or paralysis, or drowsiness (provided no hypoglycemia)<br>Resting pulse rate > 150 /min (adjusted for fever)<br>Fever ≥ 39.5 °C (rectal) and unresponsive to antipyretics > 4 hours	Give oxygen<br>IV Phenobarbitone<br>Antipyretics<br>Start IVIG - 2g/kg in two divided doses<br>Check: FBC, CRP, blood sugar, and consider lumbar puncture<br>Vital Signs Monitoring every 1-3 hours for > 6 hours
Grade 3	Serious complications in CNS or Cardiopulmonary systems:<br>Pulse >170 /min<br>Profuse sweating<br>Stage 1 hypertension (SBP > 95th centile for age)<br>Respiratory abnormalities: tachypnea, labored breathing<br>Muscle hypertonia<br>Coma (Glasgow coma score < 10)	Oxygenation<br>Consider need for ventilation<br>IV Phenobarbitone and IVIG<br>Milrinone if stage 2 hypertension (SBP > 99th for age + 5 mm Hg)<br>Dobutamine if HR> 170 bpm<br>Consider additional fever control measures<br>Invasive blood pressure monitoring<br>Check: FBC, CRP, blood sugar, and Consider lumbar puncture<br>Vital signs monitoring: every 30-60 minutes for 26 hours
Grade 4	Severe complications:<br>Acute pulmonary edema<br>Cardiac collapse	SpO2 <92% with cannula oxygen 6 litres/min)<br>Respiratory arrest or gasping respiration<br>Intubation and ventilation<br>IV Phenobarbitone<br>Dobutamine; Fluid challenge<br>Antipyretics,<br>Access CVC plus invasive BP monitoring<br>Vital signs monitoring: every 15-30 mins for 26 hours

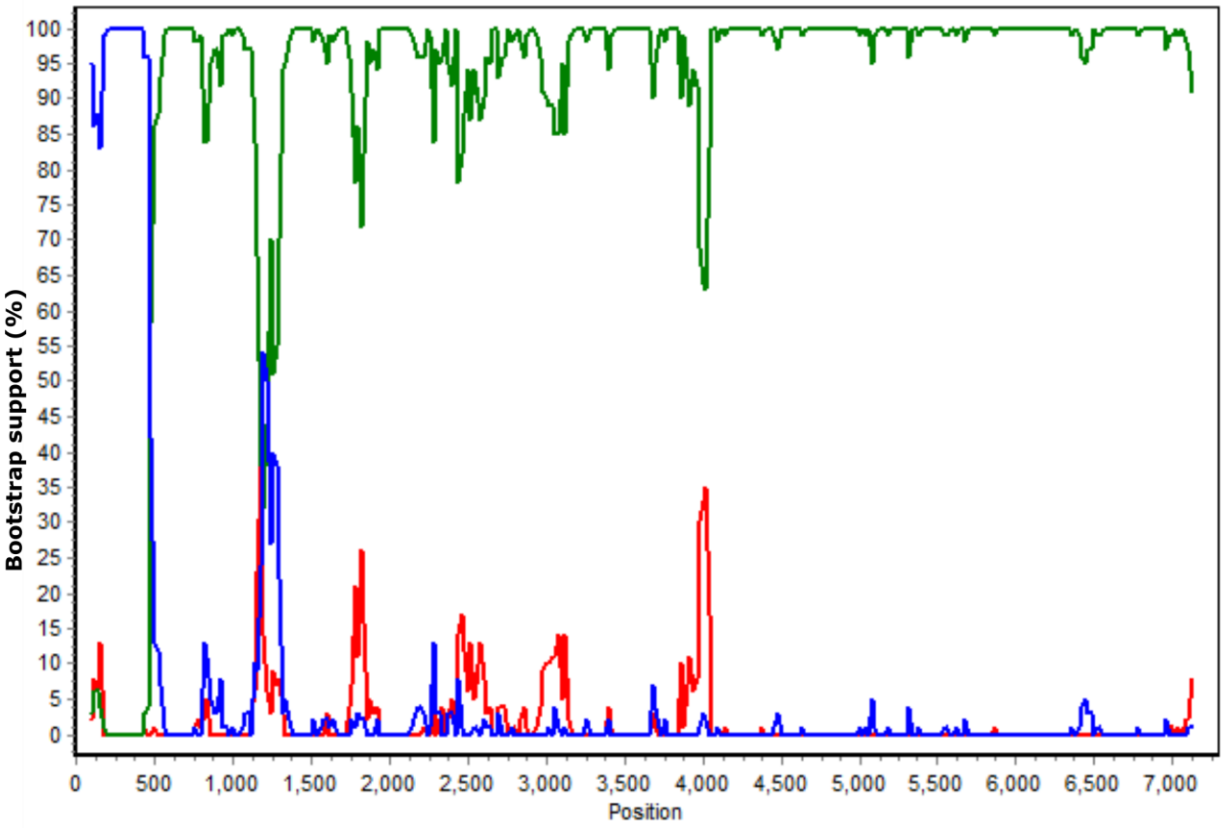

Supplementary Figure 1: Bootscan analysis showing the recombinant CVA16

Note Supplementary Figure 1: Green color indicates the recombinant CVA16 strain obtained from the present study, while red and blue colors indicate reference strains (GenBank accession numbers: KX768156 and KF193624, respectively).

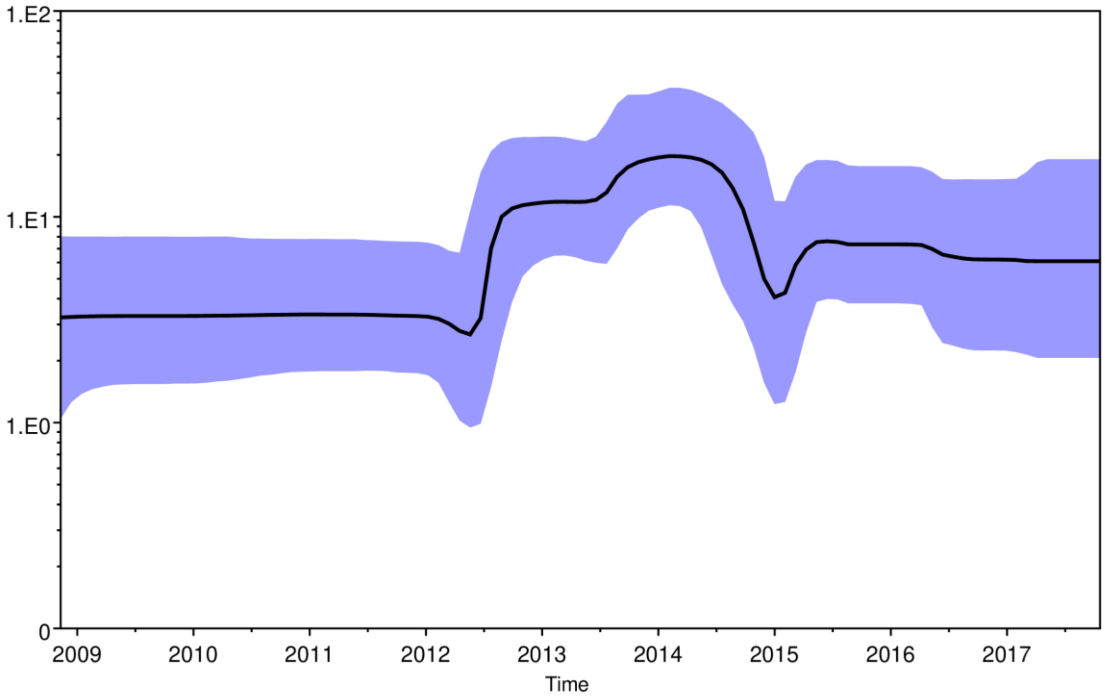

Supplementary Figure 2: Estimated relative genetic diversity of CVA16 in Vietnam by Bayesian skyline plot using CDS dataset

Note to Figure 2: The analysis was done for 68 CDS. The estimated substitution rate was 4.6 $x10^{-3}$ (4.21 – 5.02 $x10^{-3}$) substitutions per site per year. The estimated time to most recent common ancestor was Apr 2008 (Aug 2007 - Oct 2008)

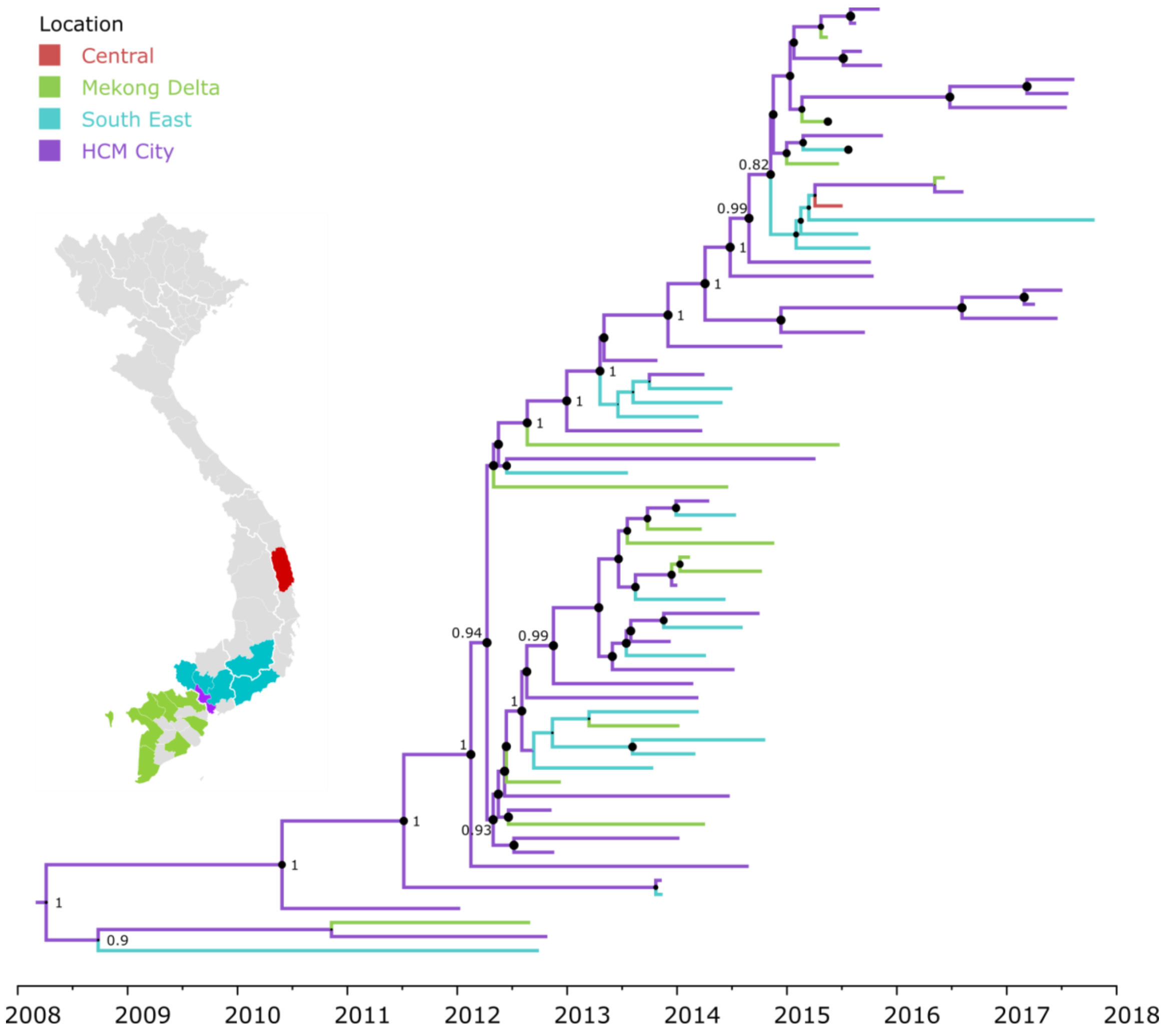

Supplementary Figure 3: CDS based maximum clade credibility trees demonstrating the phylogeography of CVA16 isolates in Vietnam
